# Ethical dilemmas concerning autonomy when persons with dementia wish to live at home: a qualitative, hermeneutic study

**DOI:** 10.1186/s12913-015-1217-1

**Published:** 2016-01-19

**Authors:** Kari Lislerud Smebye, Marit Kirkevold, Knut Engedal

**Affiliations:** 1 Lovisenberg Diaconal University College, Lovisenberggt, 15B, 0456 Oslo, Norway; 2 Institute for Health and Society, Faculty of Medicine, University of Oslo, Blindern, P.B. 1130, 0318 Oslo, Norway; 3 Institute of Public Health, Aarhus University, Aarhus, Denmark; 4 Norwegian Centre for Aging and Health, Vestfold Health Trust, 3130 Tønsberg, Norway

**Keywords:** Autonomy, Dementia, Ethical dilemmas

## Abstract

**Background:**

Caring for people with dementia living in their own homes is a challenging care issue that raises ethical dilemmas of how to balance autonomy with their safety and well-being. The theoretical framework for this study consisted of the concepts of autonomy, beneficence, non-maleficence, paternalism and from the ethics of care. The aim of this study was to explore ethical dilemmas concerning autonomy that were identified when persons with dementia wished to live at home.

**Methods:**

This Norwegian study had a qualitative, hermeneutic design and was based on nine cases. Each case consisted of of a triad: the person with dementia, the family carer and the professional caregiver. Inclusion criteria for the persons with dementia were: (1) 67 years or older (2) diagnosed with dementia (3) Clinical Dementia Rating score 2 i.e. dementia of moderate degree (4) able to communicate verbally and (5) expressed a wish to live at home. The family carers and professional caregivers registered in the patients’ records were included in the study.

An interview guide was used in interviews with family carers and professional caregivers. Field notes were written after participant observation of interactions between persons with dementia and professional caregivers during morning care or activities at a day care centre. By means of deductive analysis, autonomy-related ethical dilemmas were identified. The final interpretation was based on perspectives from the theoretical framework.

**Results:**

The analysis revealed three main ethical dilemmas: When the autonomy of the person with dementia conflicted with (1) the family carer’s and professional caregiver’s need to prevent harm (non-maleficence) (2) the beneficence of family carers and professional caregivers (3) the autonomy of the family carer.

**Conclusions:**

In order to remain living in their own homes, people with dementia accepted their dependence on others in order to uphold their actual autonomy and live in accordance with their identified values. Paternalism could be justified in light of beneficence and non-maleficence and within an ethics of care.

## Background

In Western culture, autonomy has a range of different meanings such as “… self-rule, self-determination, freedom of will, dignity, integrity, individuality, independence, responsibility and self- knowledge” ([1], p. 6). Autonomy is also identified with the qualities of intentional actions and being free from controlling influences. In medical ethics respect for autonomy is considered a fundamental principle [2]. Autonomy is a challenging issue in dementia care that needs to be understood in the context of caring for dependent persons [1, 3].

For most older people, autonomy is important for good quality of life [4, 5] as well as being able to live independently in their own homes unless limited by very poor health [6–8]. Even when institutionalized, participation in their own care is important [9–11]. Enabling people with dementia to remain involved in decision making is central to their self-determination and feelings of worth [12], in addition to promoting dignity, integrity and personhood [13–15]. Promoting autonomy is therefore considered an important aspect of person-centred dementia care [16]. Hedman [17, 18] has recently documented how people with Alzheimer’s disease who lived at home and participated in a support group strove to be independent and able, and express their sense of self.

According to Dworkin, respect for the right to autonomy is meant to protect the ability to act out of genuine preference or character or conviction or a sense of self [19]. This integrity view of autonomy allows people with dementia to construct their lives in accordance with their values and personality. The person can live true to his or her self, allowing life to continue to develop in ways congruent with their identity. Preserving those aspects of life that are of vital importance to the person needs to be emphasised for as long as possible. A crucial question raised by Dworkin is whether persons with dementia have critical interests i.e. interests that shape the person’s life as a whole and give life meaning [20]. Critical interests are different from experiential interests, which are more immediate and fluctuating experiences. Dworkin stated that for persons with dementia, only previous and pre-dementia critical interests count and they extend to all later parts of that person’s life. New critical interests are difficult to form for persons with dementia because they have lost the grip on the narrative of their lives as a whole and the sense of continuity between past, present and future [20].

Jaworska is of a different opinion and contends that persons with dementia are capable of having previous and current critical interests because they are still “valuers” who can express preferences and make choices [21]. This is apparent if the person has consistent values and has the ability to rank them. Values are the basis for selecting from available choices and without values choices are difficult to make. Therefore, respect for autonomy of the person with dementia means that family carers and professional caregivers need to help them express their values and realize their critical interests. This can mean supporting *choice autonomy* i.e. the ability to make decisions even if they do not have *agent autonomy* i.e. the ability to execute their choices [22].

Agich uses the term *actual autonomy* in order to understand what it means to respect patient autonomy, especially in long term care [1]. Actual autonomy is not primarily equated with independence and rational decision making, but with identification. What people identify with is largely unreflective yet an integral part of the decision making process. It presupposes a developed identity, the biography of a unique person and it entails the kind of life that aligns with the elderly person’s own sense of self. This understanding of autonomy implies that helpers need to be aware of identification as the basis for decision making and that persons with dementia to a large extent are dependent on their helpers to carry out their decisions. Actual autonomy is less a state than a process of being in the world with others.

With increasing severity of dementia, decision making capacity decreases [23–25]. This is a threat to autonomy and persons with dementia need help to compensate for declining abilities. In dementia care as in all health care, the principle of beneficence is the primary obligation. It entails a moral obligation to act for the benefit of others and prevent harm [2]. Non-maleficence, on the other hand, means not inflicting harm [2]. Paternalism has been conceptualized as the opposite of autonomy and can be defined as: “… the intentional overriding of one person’s preferences or actions by another person, where the person who overrides justifies this action by appeal to the goal of benefitting or of preventing or mitigating harm to the person whose preferences or actions are overridden” ([2], p. 215). Restricting autonomous actions can therefore be justified on grounds of beneficence and non-maleficence.

Paternalism is thus a relevant issue in dementia care and may involve *soft paternalism* where helpers interfere by gently persuading or acting in such a manner that they do not let persons make poor choices or they protect them against the potentially harmful consequences of their own stated preferences or actions [2]. Helpers strive to respect their autonomous wishes by influencing decisions that lead to choosing the least restrictive alternative. *Hard paternalism* involves interventions intended to prevent or mitigate harm to or to benefit a person, despite the fact that the person’s risky choices and actions can be informed, voluntary and autonomous [2]. With hard paternalism, others impose their conception of the person’s best interests on them, deny them due respect and do not give them the opportunity to influence decisions even if they might be capable of doing so.

The question of autonomy in dementia care is especially challenging in light of how vulnerable people with dementia are when living at home [26–28]. They are perceived to be at risk for problems with nutrition, falls, personal hygiene, drug management, fire hazards, getting lost, financial fraud [29–31] and social isolation [32–34]. These risks threaten autonomy. De Witt et al. [35] found that people with dementia of mild and moderate degree had risk awareness and acknowledged their limited time for living at home. They wished to postpone the time for moving to an institution for as long as possible. Family carers and professional caregivers are confronted with the need to minimalize harm (non-maleficence) and actively promote their wellbeing (beneficence).

Caring for people with dementia living at home can create ethical dilemmas of how to balance autonomy with their safety and wellbeing [27, 36, 37]. A dilemma can be defined as “(a) a difficult problem seemingly incapable of a satisfactory solution or (b) a situation involving choice between equally unsatisfactory alternatives. An ethical dilemma arises when values and moral positions or claims conflict with one another” ([38], p. 7).

The research on ethics in the care of older people has received insufficient attention and ageist attitudes are believed to be a main reason for this [39]. In a literature review by Suhonen et al. [40] they identified empirical studies focused on specific concepts such as patient autonomy, informed consent and integrity underlying ethical dilemmas during decision making mostly within an institutional setting. Rees et al. compared nurses’ perceptions with those of older people and their relatives, revealing that nurses underestimated the size and scope of ethical issues in the care of older people [39]. A study by Persson and Wästerfors found that professional caregivers trivialize older people’s complaints and allowed them to influence their daily activities only if it did not conflict with procedures in the institution [41]. Helgesen et al. [42] conducted a study in a special care unit for persons with dementia where prerequisites for patient participation were analysed. In addition to the patients’ mental capacity, other important factors were the commitment and educational level of the staff and organizational conditions such as leadership and the culture of care. This led the authors to ask whether patient participation is a losing principle in institutional care of persons with dementia. Research studies have also addressed clinical situations such as nutritional and feeding problems [43] and use of restraints [44]. The few studies that have explored ethical challenges among older patients’ family carers are mostly related to surrogate decision making [40].

In their literature review Suhonen et al. [40] documented that research carried out in home care and sheltered housing was scarcely represented. Ethical issues concerning people with mental disorders (including dementia) living in the community have been neglected [45]. This is a concern at a time when the older population and the number of people with dementia increases and more older people will be cared for in their own homes [46]. In Norway, for example, more than half of those with dementia live at home and the public policy is to reduce institutional care in favour of providing more home care and day care centres [47].

In conclusion from our review of the literature, there is no strong tradition of research in ethical dilemmas related to autonomy in dementia care and in particular related to persons with dementia living at home [48–50]. This study contributes by illuminating the ethical dilemmas involved when persons with dementia wish to live at home and adds new insights because as far as we know no comparable in-depth studies have been conducted. Empirical cases were analyzed within a theoretical framework consisting of the concepts of autonomy, beneficence, non-maleficence, paternalism and perspectives from the ethics of care.

### Aim

The aim of this study was to explore ethical dilemmas concerning autonomy that were identified when persons with dementia wished to live at home.

## Methods

This Norwegian study had a qualitative, hermeneutic design [51, 52] and was based on nine cases. Each case consisted of a triad: the person with dementia, the family carer and the professional caregiver. In all there were 27 participants.

### Participation and research context

Inclusion criteria for the persons with dementia were: (1) 67 years or older (2) diagnosed with dementia (3) Clinical Dementia Rating score 2, i.e. dementia of moderate degree (4) able to communicate verbally and (5) expressed a wish to live at home. Age 67 was chosen because this is the common retirement age in Norway.

Twenty-six older persons were asked to participate in the study and the main reasons for not being included were: no diagnosis, did not wish to participate or their family carer thought it would be too stressful.

Diversity was promoted through purposive sampling. Nine persons with dementia participated; two were men. Three persons lived independently, two lived with family carers and three had moved to sheltered housing. One person was in a nursing home but strongly wished to move home again and therefore met the inclusion criteria. Their age varied from 82 to 88 years, the mean age was 83.

The main family carers and professional caregivers registered in the records of the person with dementia were included in the study. The group of family carers consisted of three spouses, two siblings, two adult children, a daughter-in-law and a niece. Three family caregivers were men. The professional caregivers consisted of two registered nurses, six enrolled nurses and a nurse aid, all women (Table 1).

**Table 1:** Sample: persons with dementia, family carers and professional caregivers

Case	Residence	Services	Family carer	Professional caregiver
Mr A	Nursing home	Special dementia unit	Spouse	Enrolled nurse
Mrs B	Flat – lived alone	Home nursing 3 times/day; day centre for persons with dementia 5 days/week	Sister	Registered nurse
Mrs C	Sheltered housing	Home nursing 2 times/day; day centre 4 days/week	Daughter-in-law	Enrolled nurse
Mrs D	Sheltered housing	Home nursing 3 times/day; day centre 4 days/week; house cleaning and laundry 1.5 h/ fortnight	Son	Enrolled nurse
Mr F	Flat – lived with wife	Day centre 2 days/week	Spouse	Enrolled nurse
Miss G	House – lived alone	Home nursing 3 times/day; day centre 2 days/week; house cleaning 1 h/week; meals-on-wheels 3 times/week; voluntary visitor weekly	Brother	Enrolled nurse
Miss H	Sheltered housing	Special dementia unit	Niece	Nurse aid (no qualifications)
Mrs I	House – lived alone	Home nursing 2–3 times/day; day centre for persons with dementia 5 days/week; housecleaning 1.5 h/ fortnight	Daughter	Enrolled nurse
Mrs J	House – lived with husband	Day centre for persons with dementia 2 days/week	Spouse	Registered nurse

### Data collection

A semi-structured interview guide with open-ended questions was used in interviews to enable family carers and professional caregivers to answer more freely [53]. They were asked to express how they felt about their relationships with the person with dementia, how they influenced decisions about health care and their experiences of collaboration and coordination of services. They were asked to expand on any ethical dilemmas they experienced. The interviews lasted approximately one hour and were audio-recorded and transcribed verbatim. Field notes were written as soon as possible after participant observation of interactions between persons with dementia and professional caregivers during morning care or activities at a day care centre. Sensitizing concepts from the theoretical framework gave direction to the observations and gave contextual understanding of each case [53]. Reflections on what had been observed were also registered. Because of the dementia trajectory, all data in each case were collected in the course of 1–2 days. Data were collected from October 2007 to January 2009.

### Ethical considerations

Staff in three municipalities were informed about the study and asked to identify persons meeting the inclusion criteria. Persons with dementia were asked to participate after receiving information which could be reread to compensate for deficits in short-term memory. They were informed that participation was voluntary, that they could withdraw at any time and their anonymity was assured. Caregivers then asked them if they were willing to participate in the study and written consent was obtained. Even though they might have felt obliged to consent when asked by a caregiver on whom they were dependent, it was better that they were asked by a known and trusted person as this reduced anxiety [54]. Family carers were asked to consent to the participation of the person with dementia and their own participation.

Therefore, persons with dementia gave written consent and family carers also gave written consent concerning their participation. In addition family carers gave written consent on their own behalf. On the observation day persons with dementia were asked again if they still consented to participate to ensure process consent [55]. This study was conducted in compliance with the Helsinki Declaration and was approved by the Regional Committee for Medical Research Ethics in Norway, South Eastern Region (Reference number S-0718a) and Norwegian Social Science Data Services (Project number – 17352).

### Analysis

All the data in the nine cases were first analysed by reading the text from the interviews and field notes thoroughly to get a sense of the whole. A summary of each case was written and a deductive analysis [51] was conducted based on the given definition of an ethical dilemma [38]. All ethical dilemmas were identified and listed. Dilemmas centred on autonomy were then studied in greater detail and sorted into three main catagories of ethical dilemmas. For example “Installing technical devices without informed consent” was sorted under the main dilemma: “When autonomy of the person with dementia conflicts with the family carers’ and professional caregivers’ need to prevent harm (non-maleficence)”. Then each case was reexamined to check that all the ethical dilemmas pertaining to autonomy had been included. This helped to finalise the nature of the ethical dilemma. In this way the text was reviewed from the parts to the whole and back again until the ethical dilemmas were clarified in this hermeneutic process. Two aspects seemed to be common to all cases: autonomy based on critical or experiential interests and choice and agent autonomy.

Finally, the interpretation and comprehensive understanding of the autonomy-related ethical dilemmas were based on perspectives and discussions derived from central concepts in the theoretical framework.

## Results

The analysis revealed three main ethical dilemmas. First, the autonomy of the person with dementia could conflict with the family carer’s and professional caregiver’s need to prevent harm (non-maleficence). Second, the most common ethical dilemma seemed to be when autonomy of the person with dementia conflicted with what family carers and professional caregivers thought would be best to promote the wellbeing and interests of the person with dementia (beneficence). It was not always straightforward to separate the principles of beneficence and non-maleficence; sometimes these partially overlapped. Third, a dilemma occurred when autonomy of the person with dementia conflicted with the autonomy of the family carer.

Cases were selected to exemplify and illuminate these ethical dilemmas. Conflicting values and claims were described to illustrate how difficult it could be to find satisfactory solutions or to choose between equally unsatisfactory alternatives. Possible consequences were described and in some cases it was only possible to assume what might happen and offer analysis-based opinions.

### The autonomy of the person with dementia conflicted with the family carer’s and professional caregiver’s need to prevent harm (non-maleficence)

Persons with dementia are vulnerable and at risk for many problems of which they may or may not be aware. The ethical dilemma in this category was the value of autonomy versus the need to prevent harm and distress in accordance with the principle of non-maleficence. This concerned major issues such as the person with dementia deciding where to live as well as in minor everyday issues.

In situations where autonomy conflicted with security, the family and the professional caregivers were very aware of how vulnerable the persons with dementia were. They wanted to respect their autonomy but saw it as essential to take safety measures and had to strike a balance between the principle of the person’s autonomy with the principle of non-maleficence. At times paternalism was called for and they provided a web of relationships that formed a safety-net surrounding the person with dementia. Assessing the home situation on a regular basis was necessary to make required adjustments to prevent physical as well as mental and emotional harm.

The following case illustrates how difficult it can be to solve the dilemma when the principle of autonomy conflicted with the principle of non-maleficence in morning care:

*Mrs I was a widow who wished to live in her house with a scenic view and where she had lived for fifty years with many happy memories of family life. Staff from the home nursing services came two to three times a day to help her with her personal hygiene, to remind her to take her medication and prepare meals. Twice a week a bus came to bring her to the day care centre. Her daughter lived in the same town and visited or contacted her by phone daily. In the course of the last two years the daughter had only met the professional caregiver twice. In the interview the professional caregiver said she was confused about what to do when she found Mrs I in the morning fully dressed in bed under her covers. She asked Mrs I if she had forgotten to undress before going to bed the night before or if she already had been to the bathroom and washed. Mrs I said she did not need to wash as she had been up early and lay in bed because she was cold. The professional caregiver doubted this but said she felt it would be “disrespectful” if she insisted on following her to the bathroom to help her wash. Consequently, she accepted Mrs I’s explanation and carried on doing other tasks. In contrast Mrs I’s daughter described how she at times successfully helped her mother shower. Mrs I had faecal incontinence and had often had urinary tract infections. There was no written care plan providing specific directions for how morning care was to be carried out. When close to her, an unpleasant odour could be detected. (From interview with the professional caregiver, interview with the daughter, field notes and patient records)*

The ethical dilemma in this case was that the professional caregiver had to choose a course of action. Should she be guided by the principle of autonomy and let Mrs I decide by taking her word for it when she said she did not need help for morning care? Or should she be guided by the principle of non-maleficence, attempting to persuade Mrs I to follow her to the bathroom and if necessary adopt a paternalistic approach and wash her? These were equally unsatisfactory alternatives.

The consequences of respecting Mrs I’s autonomy would be to continue preparing her breakfast, thus accepting that she had already washed. This could lead to harm since it is very likely that Mrs I once again would get a urinary tract infection, possibly with further complications and especially if this happened repeatedly. When Mrs I chose not to maintain basic personal hygiene, other consequences could be that people made comments and withdrew from her at the day care centre. This could in turn threaten her dignity and personhood.

On the other hand, if the professional caregiver had insisted on following her to the bathroom, Mrs I could have felt insulted as the caregiver would then be demonstrating that she did not believe that what Mrs I said was correct. If the professional caregiver more or less persuaded Mrs I to wash, using soft paternalism, the consequences would be to prevent infection and promote wellbeing, thus justifying overriding her wishes. If things had come to a head, the use of force could have led to detrimental consequences. In the case of a serious infection, hospitalization or moving to a nursing home could be the last resort.

There were no good solutions to the problem. The professional caregiver was sensitive to Mrs I’s needs and believed she showed her respect by accepting that what she said was correct. During the interview the professional caregiver said she wished she had more knowledge of dementia so that she could understand and communicate more effectively with Mrs I. Because there was no collaboration with the daughter, she did not know how the daughter was able to shower her mother successfully. A heavy workload was another barrier to giving quality care and the professional caregiver said that she was more efficient when she went on to preparing breakfast rather than taking on the more time-consuming task of reassessing the situation and coaxing Mrs I to accept help with her personal hygiene.

With the progress of dementia it can be important to be aware of the ethical dilemmas between autonomy and non-maleficence when managing care to enable a person with dementia to live at home as long as possible. Here the pivotal role of the professional caregiver and the necessity of varied services and tailored care were demonstrated but the dilemma was not adequately addressed. With closer collaboration between the family carer and professional caregivers, and increased competence about dementia, the dilemma could probably be handled in a better way.

A similar *potential* ethical dilemma was identified in the following case; raising the question of how long it was possible for Miss G to live alone in her home (choice autonomy) despite the risks involved and which increased as dementia progressed. In the interview the professional caregiver said that it meant everything to Miss G to be able to live in her house and the home nursing services wished to support her providing agent autonomy. The professional caregivers and the family wished to safeguard her and protect her from harm.

*Miss G lived alone in a house built by her father where she had lived all her life. She lived in a working-class neighbourhood with strong local traditions and Miss G was very conscious of her class and cultural identity. When her father became ill, she cared for him in her home for many years. She stated very clearly that she wished to continue living there and that she could manage with a little help.*

*Her brother considered a nursing home to be the best alternative for her but he said: “Because of everything she has done for our father, we will not push her too hard. She can have it her way!” The general practitioner, who made regular home visits, monitored the effect of the dementia medication and had until now concluded that she was mentally competent to decide where she wished to live.*

*Home nurses visited her three times a day and a home help aide came weekly to help with housecleaning, laundry and grocery shopping. She received meals-on-wheels three days a week. Two days a week she enjoyed attending a local day care centre where she met friends and neighbours, did handwork and went to the hairdresser. In addition a voluntary visitor came once a week.*

*Her brother phoned her to remind her of which days she was to attend day care centre, took care of her financial affairs and arranged for a neighbour to do garden work. The professional caregiver had regular meetings with Miss G and her family so that they could negotiate how to support her.*

*The professional caregivers were concerned about safely issues and arranged for technical devices to be installed. Miss G fell several times, was unable to get up and needed an alarm button to summon help. Sometimes she forgot to turn off the coffee pot so they installed a new one that automatically turned itself off after a certain length of time, minimizing the fire hazard. She used to lock her front-door with a chain because she then felt safe but the professional caregiver had this removed in spite of her protests. In case of her falling again or the possibility of fire, helpers needed to be able to enter the house.*

Miss G was partially aware of the risks involved and she had told the professional caregiver that she considered these risks worth taking (from interview with the professional caregiver). Potential risks were identified and steps were taken to protect her, for example by installing technical aids. At first she had refused to have them installed (from interview with the professional caregiver). This raised new ethical issues about informed consent, surveillance and curtailing freedom as the professional caregiver was not convinced that Miss G really understood what this was all about even though she had tried to explain several times that this was necessary for her safety. Taking (hard) paternalistic action was necessary in order to secure her autonomous decision of living at home. The professional caregiver said that she needed to be sure that Miss G was as safe as possible, otherwise she would worry about her and have a guilty conscience if anything harmful happened. Later during the researcher’s (KLS) home visit, Miss G said she had now accepted these technical aids and felt safe.

### The autonomy of the person with dementia conflicted with the beneficence of family carers and professional caregivers

The overall impression was that ethical dilemmas concerning autonomy and beneficence were the most common. As dementia progressed, decreased cognitive and volitional capacity made it difficult for persons with dementia to determine what their best interests were and their decision making was impaired. They were dependent on informal care from their family members who were committed to help them and also formal care from professional caregivers who had knowledge of dementia and the necessary skills to help them. In the following case interpersonal discontinuity amongst service providers and sparse or no biographical information on the person with dementia made matters difficult. Differences in the assessment and understanding of the person’s needs hampered continuous negotiation and collaboration between involved parties. Thus, the ability to adjust to what the person had to relinquish and what he or she could still hold on to, was hampered.

This case is an example of a complex situation where the family carer, motivated by beneficence (and non-maleficence) took measures to secure the well-being of Mrs C, believing this was in accordance with her wishes:

*Mrs C lived alone in her home on a farm with her family living close by. In winter she worked hard to keep heaters burning. She had to chop wood and risked slipping on an icy path when she carried wood from the shed. Grocery shopping and eating nutritious meals were another problem. Her family thought it would be better for her to move to a sheltered housing complex with electrical heating. They provided her with information, arranged for her to see her new flat and applied for a care package that they thought she needed. Mrs C made an autonomous decision to move, signed the necessary papers and moved in. The professional caregivers saw to it that she got up in the morning, ate her meals and attended the day care centre. They tied coloured scarves on the stairs as cues to lead her to her own front door as she sometimes was unable to find her way in the large housing complex. Mrs C was on her own most afternoons and evenings. After some months she changed her mind and wanted to return home, phoning her family frequently to say she was anxious, afraid of being alone and that she no longer saw any reason for living. On one occasion she walked many miles back to the farm. Her daughter-in-law said the professional caregivers did not have updated information on Mrs C until she told them about the present situation. The daughter-in-law was very worried and without informing Mrs C, she decided to apply for placement in a special care unit (SCU) in a nursing home and signed the application herself (from interview with family carer).*

The ethical dilemma in this case is the conflict between the autonomy of the person with dementia versus the beneficence of the family. Mrs C wanted to return to her home on the farm. The family, in this case the daughter-in-law, did not believe this would ensure Mrs C’s wellbeing and considered a placement in a nursing home SCU a better solution. She thought this would be to her benefit as she then would have more company and hopefully not be as anxious and depressed. Before moving to sheltered housing, the family carer had done everything possible to ensure informed consent and autonomous decision making before Mrs C decided to move. After moving, Mrs C’s expectations had not been met and she became anxious, depressed and disorientated. Mrs C changed her mind, stating that she wanted to return home again. This raised the question of whether her decisions were based on critical or experiential interests. Deciding to return to her home was based on previous critical interests; she was also motivated by experiential interests such as hoping to feel safe and secure there. The family carer used soft paternalism to persuade Mrs C to move to sheltered housing in the first place but when applying for a placement in the SCU she used hard paternalism to justify her decision which she thought benefitted Mrs C.

For Mrs C, a possible consequence of remaining in the sheltered housing could be that she had wandered off and gotten lost or that she became even more depressed. Returning to live on the farm was no alternative as she was no longer capable of coping with the physical demands of living in an old farm house and taking care of herself even with substantial help. Her family seemed to be strongly obliged to securing her services from the municipality rather than taking her in to live with them.

Being placed in a nursing home SCU could perhaps lead to detrimental consequences for the relationship between the family carer and Mrs C when she realized that her daughter-in-law had taken action on her behalf without her knowledge. On the other hand, Mrs C could have been relieved that the burden of decision making had been taken from her and that arrangements had been made so that she felt more secure and less alone. In this case the professional caregivers did not seem to be aware of how much support Mrs C needed to feel safe and secure in her present situation.

The family carer found it necessary to draw on the principles of both beneficence and non-maleficence, making it difficult to separate the two. She wished to promote the well-being of Mrs C but this was closely linked to avoiding harm and preventing distress.

### The autonomy of the person with dementia conflicted with the autonomy of the family carer

The ethical dilemma in this category consisted of conflicting moral claims of autonomy where there was no satisfactory solution to the dilemma. In general, it seemed to become more difficult to find satisfactory solutions in families with strained realtionships where the autonomy of the person with dementia was in conflict with the autonomy of the family carer as in the case of Mr and Mrs A. On the other hand, affection and feeling responsible for the well-being of the person with dementia lessened conflicts between the person with dementia and their family carer.

In general the persons with dementia in this study appeared to think that living in their home was a reasonable claim but according to the family carers they did not always understand the consequences for their families. Another observation was that the person with dementia could have partial insight and was grateful for the help they received. Ambivalent family carers wanted to or felt it was their duty to help but said that caring responsibilities were sometimes more than they could handle since their own health deteriorated. The person with dementia was dependent on the family carer to be able to remain living in their home and it seemed to be up to the family carer to make the final decisions. This also mirrored the power imbalance in the relationships.

The following case illustrates the ethical dilemma that ensued when the autonomy of the person with dementia was in conflict with the autonomy or self-interests of the family caregiver. Whose autonomy was to be given the highest priority?

*Placement in a nursing home SCU was arranged for Mr A (MMSE = 23) as the result of a critical incident. This happened when a bus from the day care centre came to pick him up. He was totally unprepared and had angrily grasped his wife’s wrists, demanding to know why she had made decisions about the day care centre without informing him. His wife used this incident to underline his need for being in a SCU. She showed the administrator of the nursing home the black and blue marks on her wrists as proof of his physical abuse. She was aware of having provoked him by not informing him about the day care centre. She also admitted to telling him white lies about going away for treatment for her own medical problems so that he would accept a short-term stay in the SCU. Caring responsibilities had left her exhausted and she felt that she could not take any more and was desperate to find a way out of the situation. Even though the spouses had not always been on the best of terms, she still felt a strong obligation to help him. (Data from interview with Mrs A).*

*At the SCU Mr A repeatedly said he wanted to go home. At times he was aggressive and restless which the staff attributed to his dementia without collecting data to help them understand what had happened before he was admitted. They discussed the need for medicating him. Mr A had not yet been assigned a primary caregiver on the unit. (Data from interview with staff on the SCU).*

A main problem in this case was that Mr A had not been involved in decision making; he was denied the right to choice autonomy. As he lacked agent autonomy, he was dependent on help from his wife. A precondition for being able to influence decisions about the future was being better informed about the extent of his wife’s health problems and how this made caring for him a demanding task. With his MMSE score he most likely had the cognitive capacity for partial understanding of his own situation. His aggression could be understood as a legitimate response to situations he was unprepared for and contrary to his wishes. Being accused of abusing his wife probably added to his frustration and medicating him would not have solved the underlying problems.

In the interview with Mrs A, she said she felt that her autonomy and her own sense of self was threatened by her caring responsibilities. Her own health problems made matters worse. In fact she was overwhelmed by the situation and she found little time or opportunity for her own self-interests. She said she was a victim of her circumstances and unable to lead the kind of autonomous life she wanted. Yet she expressed concern for her husband’s well-being and in her opinion she had done her utmost to help him, thus justifying her paternalistic approach. In a worst case scenario, she said she might have been the one abusing her husband. However, she did not seem to be aware of the negative consequences of transferring her husband to the nursing home without informing him and against his will, and that by doing so it would not improve her relationship with her husband. She had also told “white lies” and felt guilty and worried about her reputation and what other people thought of her. This reflected her ambivalence when faced with living up to societal norms.

Finding a satisfactory solution to the ethical dilemma in this case was difficult. Because it was impossible to honour the autonomy of the person with dementia and the family carer simultaneously, the staff was left with the challenge of helping Mr A understand his wife’s situation and promoting optimal autonomy for him in the special care unit. However, there was no evidence of the staff being aware of the dilemma as this was not mentioned in the interview with the professional caregiver nor registered as a topic of conversation on the ward (from field notes).

## Discussion

Autonomy is usually associated with independence. Nonetheless it is a fact that persons with dementia even though they wish to be autonomous and remain living at home, will in time become increasingly dependent on others. How can autonomy then be understood and promoted? The ethics of care offers a broad approach that can give new insights and advance comprehensive understanding of the ethical dilemmas in this study. In dementia care paternalism is a relevant issue and a pertinent question is if this is beneficial or detrimental in autonomy-related dilemmas.

### Combining dependency and autonomy in dementia care

In this study persons with dementia who wished to remain living in their own homes, appeared to accept dependence on family carers and professional caregivers who made this possible. The cases presented in this study demonstrated how important it was to explore ethical dilemmas within an ethics of care emphasising the importance of relationships and communication, the particular context, the uniqueness of individuals and empathetic understanding [56–58]. Several studies document that professional caregivers who knew the person with dementia well collaborated with their families and other health professionals and built relationships important for trust and security. When necessary they negotiated to reconcile competing interests between parties [59, 60]. This coincides with how professional caregivers in this study combined empathy and professional knowledge in their continual assessments of the person’s condition, which they knew would deteriorate and they expected them to become more confused and anxious as time passed. Nevertheless, in-depth knowledge of these persons enabled them to offer more meaningful choices and allow for more risk-taking compared to others who did not know their patients as well. When persons with dementia expressed either verbally or non-verbally that their well-being was threatened or risks were difficult to prevent, the professional caregivers were in a position to register subtle cues and know when it was time to intervene. Ethical dilemmas could be resolved before a crisis developed.

Prerequisites for combining dependency and autonomy in dementia care were that family carers and professional caregivers responded to the vulnerability of the persons with dementia [61]. Within an ethics of care, sensitivity and empathy are necessary virtues but this does not reduce ethics merely to an emotional response as caring also has a cognitive dimension [62]. Professional knowledge is required to be able to respond adequately and appropriately to other people’s needs. This requires attentiveness, responsibility, competence and responsiveness and it follows that those who care are active, committed and involved [63]. Caring is a dynamic and ongoing process requiring more than making the right decision at a certain moment. Rather, it demands continuous involvement and decision making [64].

In cases where dependency and autonomy were not so easily combined, routines and task-oriented care dominated, especially if a professional caregiver had not been assigned to the person with dementia on a regular basis. This occurred in the case of Mr A since his individual needs were not catered to and his wife did not receive sufficient support when her sense of self was threatened by caring responsibilities. Mrs I’s professional caregiver lacked professional knowledge and needed clinical supervision to be able to handle the ethical dilemmas she encountered, while Mrs C’s professional caregivers lacked information which could otherwise have spurred them to take action (from interview with family carer).

According to Agich, being dependent on others does not hinder actual autonomy where decisions are based on values people identify with and especially in those areas of life that they value [1]. “Dependencies as such do not conflict with autonomy if individuals still maintain a sufficiently adequate range of identification to sustain their personal sense of integrity and worth” ([1], p.121).

In some cases it was difficult to determine what the person with dementia actually valued and whether their wishes were based on critical or experiential interests or whether these interests were in conflict or in harmony. In the case of Miss G and Mrs I it was apparent that their wishes reflected stable and long-lasting values based on past and present critical interests. Home was a place associated with important life events that had contributed to their development as persons, vital to a sense of autonomy and continuity of self [65–67]. This aligns with Jaworska’s thoughts on being a “valuer” with the ability to prioritize values [21]. According to Holm it is necessary to trace a connection between the desire and the person’s former personality and narrative to assess whether the preferences are in character and can be called critical interests [68]. For Mr A and Mrs I there were no professional caregivers who felt responsible for collecting biographical and health data to assess their interests.

Experiential interests fluctuate and are not as stable as critical interests since the immediate situation has a greater impact on decision making. Current needs and aroused feelings can destabilise long held preferences, making decisions more unstable and people tend to change their minds more often. This is supported by the findings in the present study. Mrs C moved to sheltered housing but changed her mind and wanted to return to her farm as she felt anxious and alone. For persons with dementia, feeling at home brings with it a sense of belonging and comfort; home is a place where identity can be preserved [69, 70].

Can experiential interests become more important than critical interests? Security became more important than autonomy for Mrs C. She felt insecure in the sheltered housing and so she longed for home. To complicate matters even more, had she returned to the farm she would most likely still have felt insecure. Hence, assessing the underlying causes of her present insecurity in the sheltered housing was imperative as well as reflecting on what autonomy meant to her now that her life circumstances had changed. Holm states: “We will always need to know not only what critical interests the patient had formed, but also what the reasons are for forming these interests” ([68], p. 157).

This indicates that autonomy must be considered and balanced with other interests. Ideally it should have been possible to combine being secure and autonomous and combining critical and experiential interests. The cases in this study illustrate that it is not so easy to neatly categorize values into critical and experiential interests as the basis for decision making. Yet, using the concepts of critical and experiential interests can add to understanding of the ethical dilemmas if not always helping to solve them. Facing ethical dilemmas involves continuous assessment of values and interests.

### Paternalism can promote autonomy and justify beneficence and non-maleficence

Paternalism was an issue in the ethical dilemmas for the people in this study where autonomy conflicted with beneficence and non-maleficence. At times the professional caregivers used a hard paternalistic approach in order to avoid harm by for example installing technical devices in a home. This was done without the person with dementia fully understanding the situation or consenting. However, they justified this as a necessary measure that enabled the person to remain at home in accordance with their autonomous decision. Therefore, paternalism is not necessarily the opposite of autonomy but can actually be necessary to realize critical interests.

The cases offer examples of soft and hard paternalism. Mrs I’s caregiver could have used soft paternalism to try to persuade her that she needed help with morning care especially if she was often found fully dressed in bed. It was not difficult to understand how overcome Mrs A was about being abused by her husband or how desperate Mrs C’s family was when she no longer wanted to live. This can account for why they demonstrated hard paternalism and ignored wishes of autonomy. According to Beauchamp and Childress actions that prevent major harms or provide major benefits while only trivially disrespecting autonomy have a plausible paternalistic rationale [2]. However, the acceptance of soft paternalism runs the risk of preparing the way for (unnecessary) hard paternalism. Paternalism can thus be beneficial or detrimental for persons with dementia and usually the best alternative is to start out by using the least restrictive measures since paternalism can lead to abuse if it is taken too far [71, 72].

Not all persons with dementia are fully aware of the risks involved by choosing to live independently. They need support from others who can identify risks and prevent harm. Nonetheless, taking risks is an inherent part of everyday life and life without any risks is unimaginable. In dementia care, family carers and professional caregivers can perhaps consider it necessary to minimize risks. This can lead to restricting freedom and foregoing benefits, detrimental to a sense of autonomy. A risk-benefit assessment of protection from harm versus the loss of autonomy is necessary.

The principle of beneficence is the primary obligation in health care and is a positive duty to act for the benefit of others and to promote well-being and not merely avoid harm. The principle of non-maleficence is a negative duty to refrain from acting or inflicting harm. The two principles can be seen as two independent principles or they can overlap, combining non-infliction of harm with positively benefitting another. The findings in this study support the latter as it proved to be difficult to separate these principles when analysing the ethical dilemmas and to isolate the specific dilemmas in each case, as seen in Mrs C’s situation.

### Strengths and limitations of the study

The strengths of this study were that sensitivity to ethical dilemmas was developed through case studies with rich descriptions of particular situations and the unfolding of complexities in each case [73–75]. The concept of autonomy is open to interpretation and specification and can best be understood when tested empirically. In this study the ethical dilemmas in each case were intrinsically connected to the specific context and needed to be understood in their entirety. The aim was to describe ethical dilemmas concerning autonomy when persons with dementia wished to live at home. Raising pertinent questions can shape ideas and shift conceptions. In this way knowledge gained from reflecting on empirical examples can inform moral judgement and improve ethical proficiency in dementia care [64].

Purposeful sampling promoted diversity with cases representing differences in gender, rural/urban settings, civil status, living alone or together with family carers. The credibility of the findings was strengthened by continually focusing on the research question, especially during data collection, analysis and interpretation [76, 77]. Triangulation of data added to the rigour of the study [51, 78]. This was achieved by using different data collection methods, different data sources, investigator triangulation (all researchers contributed to the analysis and discussion) and multi- perspectives when interpreting the data within the theoretical framework. All data were collected by the same researcher (KLS). Preconceptions were scrutinized and discussed repeatedly, especially the blurring of roles of health professional and researcher. For the novice (KLS) this increased awareness of how the text was influenced during the study; an important aspect described by Gadamer [79].

A limitation of the study was the small sample. If more cases had been included, additional types of ethical dilemmas or combinations of ethical dilemmas might have been identified. The results cannot be generalized to all persons with dementia living at home, yet the study did document relevant autonomy-related ethical dilemmas. Another limitation was that the study was only carried out in a Norwegian context. In other countries where autonomy is not so highly valued, the ethical dilemmas could have been understood differently. Also, family norms vary across countries and where the welfare state is more developed, it moderates demanding family obligations and allows for more independent relationships between generations [80].

## Conclusions

The aim of this study was to explore ethical dilemmas concerning autonomy when persons with dementia wished to live at home. This study revealed three main ethical dilemmas: (1) The autonomy of the person with dementia conflicted with the family carer’s and professional caregiver’s need to prevent harm (non-maleficence); (2) The autonomy of the person with dementia conflicted with the beneficence of family carers and professional caregivers; (3) The autonomy of the person with dementia conflicted with the autonomy of the family carer. In order to remain living in their own homes, people with dementia could accept being dependent on others in order to uphold their actual autonomy. Paternalism could be justified in light of beneficence and non-maleficence and within an ethics of care.

## Abbreviations

SCU: Special care unit
MMSE: Minimal mental state examination – moderately demented 20–25 points, max. 30 points
